# Study of the response behavior of a CdS–SnO_2_ thick film for high selectivity towards propanol gas

**DOI:** 10.1039/d4ra01888e

**Published:** 2024-05-21

**Authors:** Ankit Kumar Vishwakarma, Ajaya Kumar Sharma, Lallan Yadava

**Affiliations:** a Thin Film Laboratory, Department of Physics, Deen Dayal Upadhyaya Gorakhpur University Gorakhpur U. P. India kv.ankit92@gmail.com nisaly06@rediffmail.com +91 9450253084 +91 7309094050

## Abstract

Gas monitoring devices are in demand for a rapidly growing range of applications. Metal oxide-based gas sensors have been extensively used for the detection of toxic pollutant gases, combustible gases, and hydrocarbon vapors. The sensitivity for a low concentration and observed response and the recovery times of the reported gas sensors are not satisfactory, and it needs further detailed studies. In the present work, undoped SnO_2_ and cadmium sulfide (CdS)-doped SnO_2_ thick films were fabricated using the screen-printing method to study their sensing behavior towards tested organic vapors such as acetone, propanol, and ethanol. The sensing properties of fabricated sensors were investigated for the test gases, *i.e.* acetone, propanol, and ethanol, at an elevated temperature of 473 K. It was observed that the 2 wt% CdS-doped SnO_2_ sensor showed a maximum response (78%) and was highly selective (44.6%) to propanol over acetone and ethanol. The results showed that the diminution of the SnO_2_ crystallite size with the CdS content leads to an improvement in the response of the SnO_2_ sensor for the tested gases. The microstructural properties are also correlated to the sensing behavior. The measurement showed that the CdS–SnO_2_ thick film sensor is highly sensitive. At the same time, it is more selective to propanol than the other test gases, ethanol and acetone.

## Abstract 2

In the present paper we report structural and sensing properties of undoped and CdS-doped SnO_2_ thick film sensor for detection of propanol gas at operating temperature 200 °C.

## Graphical abstract

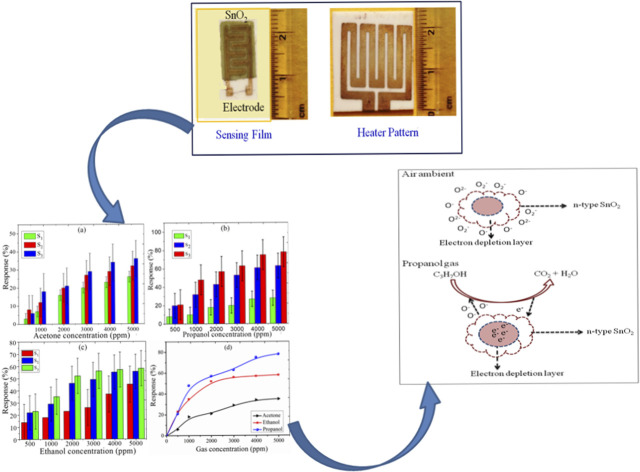

## 1. Introduction

In recent years, semiconducting oxide thin/thick films have become more attractive due to their properties such as microstructural and optical properties, high stability, and the excellent sensing devices.^1–4^ Semiconducting oxides are classified as n-type and p-type. Currently, p-type semiconducting materials are required for excellent application of the sensor. In particular, SnO_2_ is usually regarded as an oxygen-deficient n-type semiconducting material.^5^ It has many applications such as transparent conductivity, the electrode of the solar cell, gas sensitivity for gas sensor devices, photochemical and photoconductivity devices in LEDs, and gas discharge display.^6–11^ In the sensor technology, the 3S rule is applied, *i.e.*, S = sensitivity, S = selectivity, S = stability. There are various dopants used in the SnO_2_ thick/thin film sensor (such as Pd, Ni, Cd, PbO, Fe) to enhance its selectivity and sensitivity and improve its response.^12–15^ SnO_2_ is used in sensors of combustible gases including carbon monoxide detectors. In these sensors, the sensor area is heated to a constant temperature (a few hundred °C), and in the presence of a combustible gas, the electrical resistivity drops.^16^ Doping with various compounds has been investigated (*e.g.*, with CuO).^17^ Doping with cobalt and manganese gives a material that can be used in high voltage, for example; in addition, tin(iv) oxide can be doped with the oxides of iron or manganese.^18^ J. K. Srivastava *et al.*^19^ reported on the microstructural proprieties of the PbO-doped SnO_2_ sensor for the detection of methanol, propanol, and acetone. They observed that at 3wt% PbO–SnO_2_ gives the maximum response for propanol at 350 °C. They also described the optimization of the firing temperature of the doped SnO_2_ sensor for methanol and acetone detection.^12^ F. H. Saboor *et al.*^13^ presented the NO_2_ gas sensing for Pd-loaded SnO_2_ thick film gas sensors under UV light irradiation at room temperature. Xi-Tao Yin *et al.*^15^ studied the selectivity and sensitivity of a Pd-loaded Fe-doped SnO_2_ sensor for CO detection in the presence of H_2_, and showed that 10 mol% Fe, 0.2 mol% Pd gives the maximum sensitivity and selectivity to CO against H_2_. M. Choudry *et al.*^20^ reported the effect of temperature on a Pd-doped SnO_2_ thick film gas sensor and observed that the firing temperature at 800 °C gives a more selective sensor for the detection of LPG, H_2_, and CH_4_, and the reported selectivity of LPG with a maximum sensitivity of up to 87%. In a previous paper, Yadava *et al.*^21^ reported on the sensing properties of the CdS-doped SnO_2_ thick film gas sensor for the detection of methanol, and showed that the 2wt% CdS-doped SnO_2_ sensor is a suitable detector for methanol. In the present study, we report the microstructural properties and sensing response of the CdS-doped SnO_2_ thick film. The reduction in crystallite size with the addition of the lower CdS content leads to an improvement in the response of the SnO_2_ sensor for the target gases, propanol, methanol, and acetone. The microstructural properties are correlated with the sensing behavior of the undoped and doped SnO_2_ sensors. It is observed that the 2 wt% CdS doped SnO_2_ sensor showed a maximum response (78%) and was highly selective (44.6%) to propanol over acetone and ethanol.

## 2. Experimental

### 2.1. Synthesis and fabrication of the sensor

**Fig. 1:**
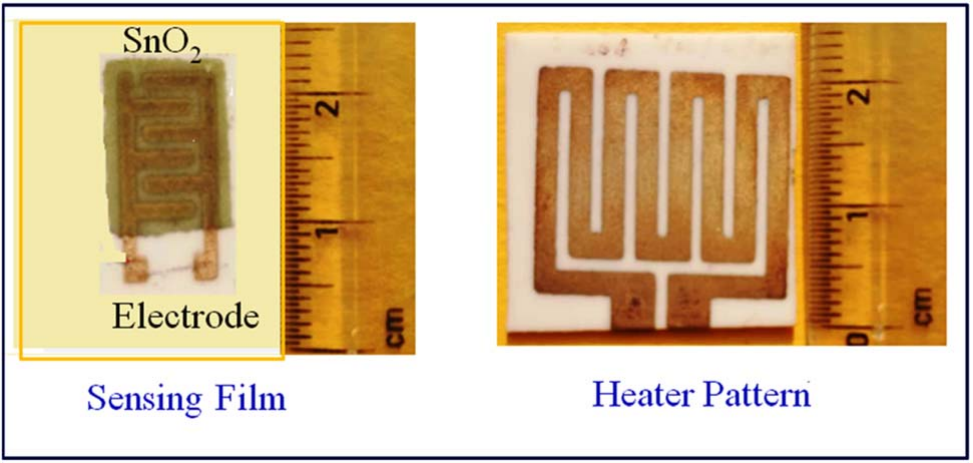
Finger electrode and heater pattern of the sensor.

In the laboratory, we take tin oxide (SnO_2_) powder and glass binder (10 wt% of SnO_2_), and mix them properly using a ball mill (Zirconia Ball Mill, Retsch) for 3–4 hours.^21^ Fine grains are then mixed with an organic binder (diethyl glycol mono butyl) and organic solvent (α-terpinol) in a ball mill for 1–2 h, which results in an undoped SnO_2_ paste. For doped pastes of SnO_2_, weighed SnO_2_ powder with glass binder (10 wt% of SnO_2_) and CdS powder (1 wt%, 2 wt%), all of these components are mixed in a ball mill, and the same organic binder and solvent are used to obtain the sensing paste. The prepared paste is screen-printed on an alumina substrate (25 mm × 25 mm) with a finger electrode pattern on the front side and a resistor heater pattern on the backside, as shown in Fig. 1.

### 2.2. Characterization and sensing setup

XRD analysis was performed on the structural and lattice parameters of the *S*_1_, *S*_2_, and *S*_3_ films. The D8-Advanced apparatus, connected with a source of Cu Kα_1_ radiation with a wavelength of 0.15406 nm, produced the XRD pattern. For the measurement of the response of the fabricated sensors, *S*_1_, *S*_2,_ and *S*_3_, a test chamber has been made in which provisions are made for the electrical connection and inlet/outlet for test gases. The connectivity for the voltage supply and resistance measurement is available through insulated gaskets on the base of the chamber. The resistance of the fabricated sensors in the air and gas environments is measured with the help of a Dual DC power supply (LD-3202) and Digital Multimeter (Aplab 107N). The response of the fabricated sensors is measured in ambient air with varying concentrations of acetone, propanol, and ethanol gases (0–5000 ppm) separately at 473 K.

## 3. Result and discussion

### 3.1. XRD

**Fig. 2:**
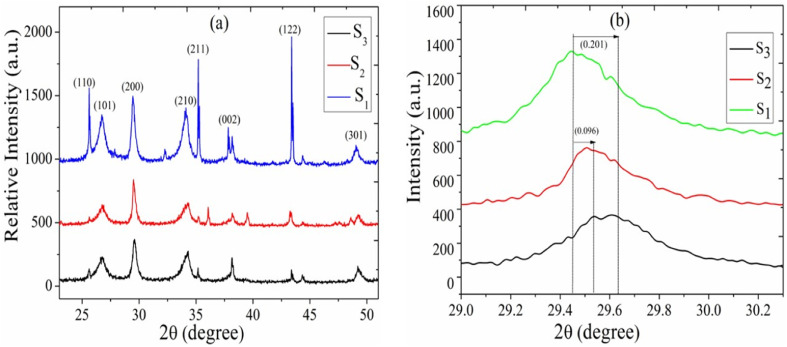
(a) XRD patterns of sensors *S*_1_, *S*_2_, and *S*_3_ (b) zoomed image of plane 200.

The experimental XRD pattern of samples *S*_1_, *S*_2_, and *S*_3_ is shown in Fig. 2. The XRD pattern of the fabricated samples has been performed in a wide range of diffraction angles from 0° to 90°. The comparative study of the XRD pattern for the sensors *S*_1_, *S*_2_, and *S*_3_ was performed in diffraction angles from 20° to 55° only. At an angle of 2*θ*, the peaks of the diffraction pattern correspond to the planes (110), (101), (200), (210), (002), (122), and (301), respectively. The polycrystalline structure of the deposited film is confirmed by these peaks and the correlating planes. If we increase the concentration of CdS in the sensors *S*_2_ and *S*_3_, modification in the XRD pattern is obtained. As we can see from the magnified figure at the plane (200) in Fig. 2(b), the peaks shift toward a higher value of 2*θ.* As the concentration of CdS increases in the sample, the peak intensities decrease and the peaks are broadening. The crystalline size can be calculated by the well-known formula given by the Debye Scherer relationship.^22,23^1
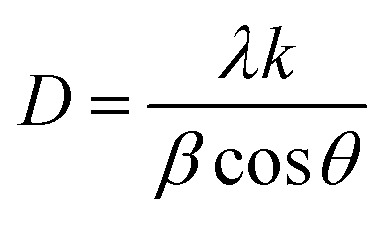
In this case, *k* = 0.94, where *λ* is the X-ray source's wavelength, *β* is the full width at half maximum (FWHM), and *θ* is the diffraction angle. The crystallite sizes of samples *S*_1_, *S*_2_, and *S*_3_ are found at 25.1 nm, 16.1 nm, and 13.9 nm, respectively. This shows that the crystallite size of the SnO_2_ films decreases with increasing CdS concentration.

To calculate microstrain, we can use the formula given below:^24^2
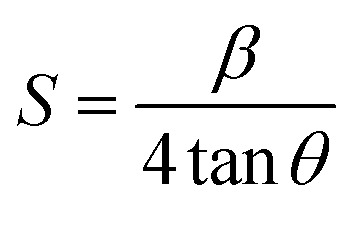


**Fig. 3:**
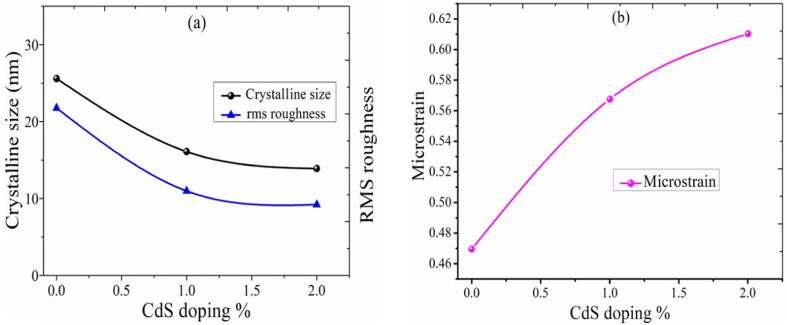
(a) Variation of the CdS content *vs.* crystallite size, rms roughness. (b) Variation of the CdS content *vs.* microstrain.

The calculated microstrain increases with the increase of the CdS concentration. The crystallite size, rms roughness, and microstrain *versus* the CdS concentration are shown in Fig. 3(a and b), and the calculated value of the microstrain is 0.47 nm, 0.57 nm, and 0.61 nm for *S*_1_, *S*_2_, and *S*_3_. Also, we observed that the crystallite size and roughness decrease with an increase in the CdS contents (Fig. 3(a)).

### 3.2. Gas sensing response

**Fig. 4:**
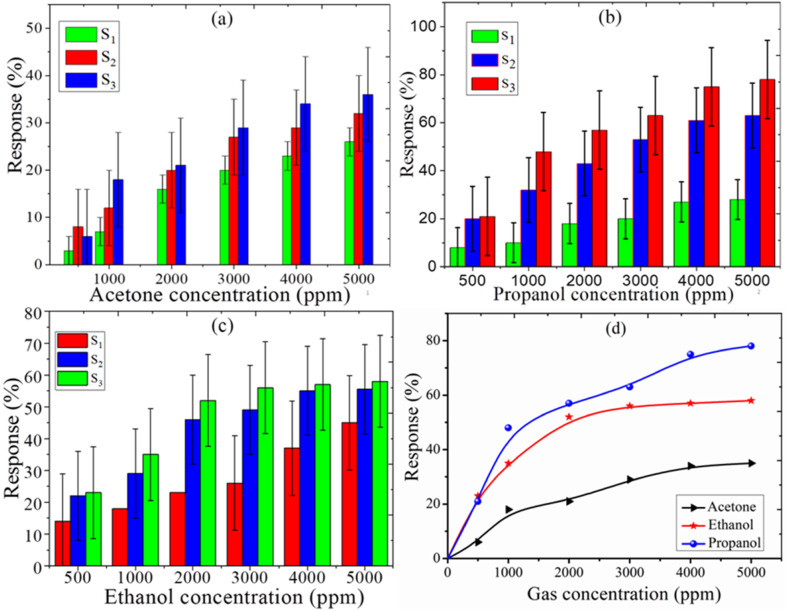
Sensing response with standard deviation error bars for (a) acetone, (b) propanol, and (c) ethanol gas for sensors *S*_1_, *S*_2_, and *S*_3_. (d) Comparative response with the standard deviation error bars of sensor *S*_3_.

The sensitivity is defined as the slopes of response *vs.* the gas concentration in ppm. Sensitivity is calculated by the following formula:^25,26^3
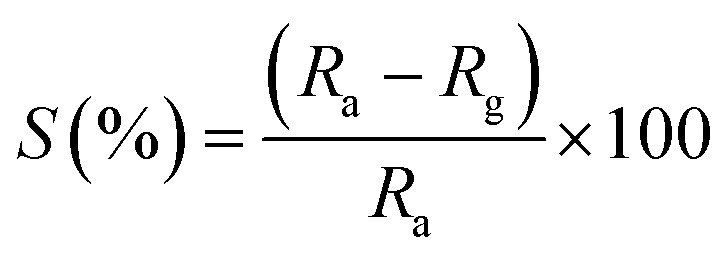
where *R*_a_ is the resistance in clean air and *R*_g_ is the resistance in the presence of vapors. The response of the fabricated sensors, *S*_1_, *S*_2_, and *S*_3_, are measured with varying concentrations (0–5000 ppm) of the target gases, acetone, ethanol, and propanol, at a fixed operating temperature of 200 °C. The response with the concentration of acetone for sensors *S*_1_, *S*_2_, and *S*_3_ is shown in Fig. 4(a). The response increases with increasing concentration of the acetone vapors. The sensitivity is 36%, 31%, and 25% for the *S*_3_, *S*_2_, and *S*_1_ sensors, respectively. It is evident from Fig. 4(b) that the maximum response for propanol was achieved with the *S*_3_ sensor (78%); it is (1.14) times that of *S*_2_ and (2.79) times that of *S*_1_. The response curve of ethanol is shown in Fig. 4(c). The sensitivity of the fabricated sensor has been reported in Table 1. Fig. 4(d) represents the comparison curve of the *S*_3_ sensor for acetone, ethanol, and propanol. It is observed that sensor *S*_3_ is more sensitive to propanol gas than acetone and ethanol. The sensitivity of propanol for sensor *S*_3_ is 1.34 times that for ethanol and 2.16 times that for acetone. Therefore, propanol has a maximum response compared to the other test gasses. The standard deviation of the response of sensors *S*_1_, *S*_2_, and *S*_3_ for acetone is 5.1, 9.7, and 11.2, respectively. The standard deviation of the response of sensors *S*_1_, *S*_2_, and *S*_3_ for propanol gas is 8.33, 16.97, and 18.87, respectively. The standard deviation of the response of sensors *S*_1_, *S*_2_, and *S*_3_ for ethanol gas is 14.85, 14.01, and 14.46, respectively.

**Table 1:** Sensing response and selectivity and transient time of the *S*_3_ sensor for propanol, ethanol, and acetone

Test gas	Response (%)			Selectivity (%)	Transient time for the *S*_3_ sensor	
	*S*_1_	*S*_2_	*S*_3_		Response time (s)	Recovery time (s)
Ethanol	45	56	58	33.91	40	70
Propanol	28	65	78	45.63	19	67
Acetone	26	32	36	20.46	45	82

### 3.3. Selectivity

**Fig. 5:**
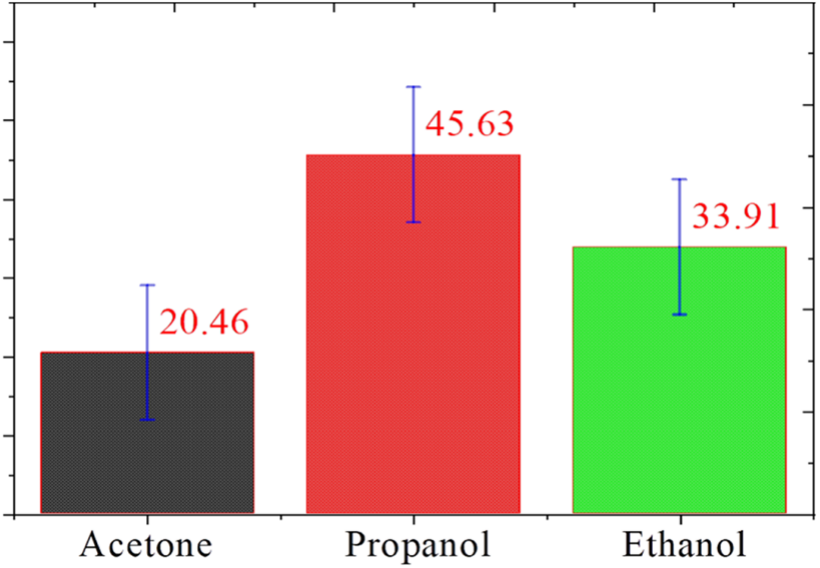
Selectivity of ethanol, acetone, and propanol gas with standard deviation error bars for sensor *S*_3_.

Selectivity is one of the most important parameters of the sensing properties of the gas sensor. The selectivity of the sensor was calculated using the following formula:^27,28^4
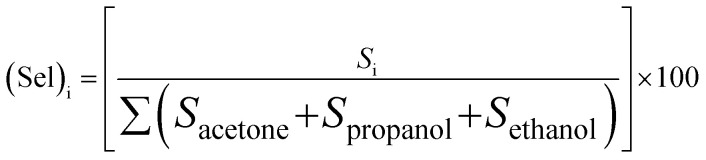
where *S*_i_ is the response of gas for which the relative is to be estimated. The responses were found to be ∼45.63%, ∼33.91%, and ∼20.46% for propanol, ethanol, and acetone, respectively, which is displayed in the histogram (Fig. 5). It is evident from Fig. 4 that propanol is more selective over ethanol and acetone. The standard deviation of the selectivity of *S*_3_ for the gases (acetone, ethanol, and propanol) is 12.59 with a median of 33.91. Recently, N. Barroso *et al.* studied the guest-induced breathing mediated selective alcohol recovery from water by MIL-88A(Fe).^29^ They explained how the flexible nature of the crystal structure of MIL-88A(Fe) impacted its adsorptive performance for the recovery of alcohol from water. They found that the results acquired in this work can open new avenues toward the rational design and potential utilization of flexible MOF-based systems, offering enhanced selectivity and sorption performance toward the challenging liquid–liquid separation.

### 3.4. Transient time

**Fig. 6:**
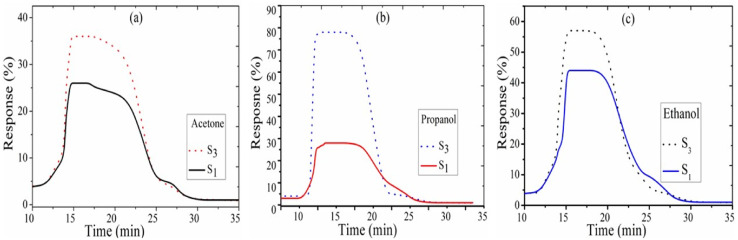
Transient time for (a) acetone, (b) propanol, and (c) ethanol gas for sensors *S*_1_ and *S*_3_.

Fig. 4 shows the sensing response *vs.* time of sensors *S*_3_ and *S*_1_ for test gases at 473 K. At time 10 min, 5000 ppm acetone was injected in the test chamber to get the maximum response, and then the chamber was opened to recover the initial state of the sensor. The time corresponds to the time required for the gas response to reach 90% of the final equilibrium value after a test gas is injected, and the recovery time is the time recorded for the gas response to decrease by 90% its maximum value when the gas sensor is exposed in ambient air.^30,31^Fig. 6(b) shows that in the *S*_3_ samples, the fast response and recovery times decrease from 31 s to 19 s and 89 s to 67 s for propanol (5000 ppm at 200 °C), respectively. The transient response and recovery curve to the acetone and ethanol sensors *S*_1_ and *S*_3_ is plotted in Fig. 6(a and c), and its value is listed in Table 1.

### 3.5. Sensing mechanism

The sensing mechanism of the fabricated sensors (*S*_1_, *S*_2_, *S*_3_) can be realized as the resistance of materials varies with oxygen molecules absorbed at the surface and with the amount of reducing gas species. When metal oxide comes in contact with air, the oxygen molecules from the ambient environment are absorbed onto the surface of the metal oxide at the grain boundaries, which trap electrons and build a barrier around each grain.^32^ The adsorbed oxygen molecules were subsequently converted to ions after capturing an electron from the conduction band. The adsorbed oxygen exists on the surface in the form of O^−^, O_2_^−^, and O^2−^ species, depending on the temperature.^33^ The chemical absorption process can be explained by the following reaction (eqn (5)–(8)):

5 O_2(gas)_ ↔ O_2(ads.)_

If the operating temperature *T* < 150 °C:

6 O_2(ads.)_ + e^−^ ↔ O_2(ads.)_^−^

If the operating temperature is 150 °C < *T* < 400 °C:

7 O_2(ads.)_ + e^−^ ↔ 2O_(ads.)_^−^

If the operating temperature *T* > 400 °C:

8 O_2(ads.)_ + e^−^ ↔ O_(ads.)_^2−^

**Fig. 7:**
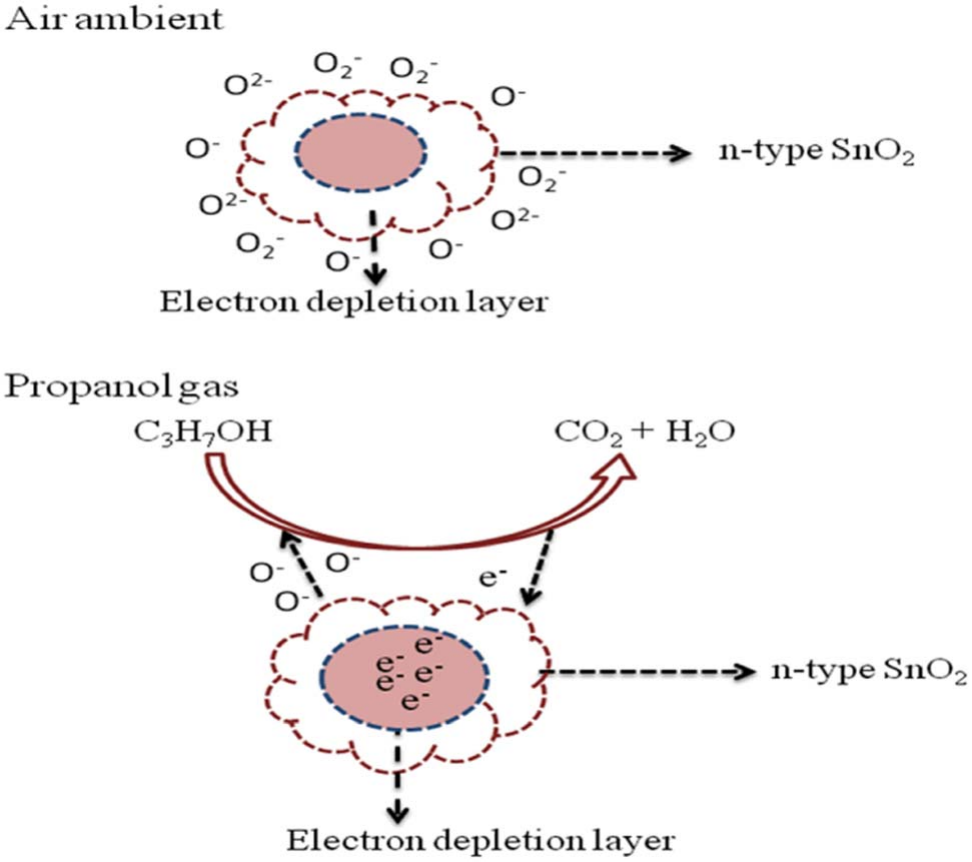
Reaction process of propanol gas in the SnO_2_ thick film.

When sensor *S*_3_ contacts with the reducing gas, the reducing gas propanol (C_3_H_7_OH) reacts with the oxygen species to produce carbon dioxide and water, and the resulting electron will return to the conduction band of the semiconductor. The possible surface reactions take place (Fig. 7):

9 C_3_H_7_OH + 9O^−^ → 3CO_2_ + 4H_2_O + 9e^−^

Among the adsorbed oxygen species (O_2_−, O^2−^, and O^−^) on the surface, the majorly atomic species (O^−^ single ionized oxygen) is more reactive, particularly at higher temperatures (150 °C < *T* < 400 °C). When the CdS–SnO_2_ sensor is exposed to the target gases, propanol, ethanol, and acetone, at an elevated temperature of 200 °C, the gas will be adsorbed on the surface of the sensor. These gases will react with the chemically adsorbed oxygen anions, particularly O^−^. The electrons are released back to the material of cadmium sulfide, resulting in a decrease in the depletion layer thickness on the surface of the sensor, and also a decrease in the surface band bending. Therefore, the change in the electrical resistance of the sensor is observed with exposure to target gases. Among the target gases, the *S*_3_ sensor showed a larger response and at the same time high selectivity, which may be due to its different molecular structures. Also, it is obvious from eqn (9) that the carrier's concentration increased, which caused the sensor resistance to be reduced. Therefore, this process will increase the carrier concentration on the surface of tin oxide, resulting in a decrease in resistance.^34^ For a definite understanding regarding the high selectivity, future work is to be done using an array of sensors and exposure to a mixture of target gases.

We compare our findings to other related work, and a comparison table of the sensing response, operating temperature, and response time for alcoholic and acetone gas is listed in Table 2.

**Table 2:** A comparison table of the sensing response, operating temperature, and response time for alcoholic and acetone gas

Sample	Test gas	Operating temperature (°C)	Sensitivity (%)	Response time (s)	References
CdS–TiO_2_	Propanol	—	63	62	25
CdS–TiO_2_	Acetone	—	71	55	27
Sb_2_O_3_–SnO_2_	Ethanol	250	73.5	—	32
Pd–SnO_2_	Ethanol	200	71	41	33
TiO_2_	Propanol	—	26	—	34
LaFeO_3_-based oxide	Propanol	100	258.4	—	35
NiO	Propanol	75	60	—	36
SnO_2_	Propanol	150	71	—	37
Pd–SnO_2_	Propanol	300	92	—	38
ZnO/TiO_2_	Propanol	—	23	10	39
SnO_2_ nanorods	Isopropanol	325	11.2	6	40
SnO_2_–Pd–Pt–In_2_O_3_	Methanol	160	320.7	32	41
Ce-doped SnO_2_	Acetone	270	50.5	—	42
Cactus-like WO_3_–SnO_2_	Acetone	360	26	—	43
CdS–SnO_2_	Propanol	200	78	19	Present work

## 4. Conclusion

Three types of sensors, namely *S*_1_ (undoped SnO_2_), *S*_2_ (1 wt% CdS–SnO_2_), and *S*_3_ (2 wt% CdS–SnO_2_), are fabricated. It is found from the XRD pattern that the crystallite size and roughness decrease with dopant CdS (0–2%) and it lies within the anemometric range. The diminution of the SnO_2_ crystallite size leads to an improvement in the sensitivity of the fabricated sensors for test gases. Microstructural properties are correlated with the sensitivity of the materials and dopants. The sensing behavior of the sensors *S*_1_, *S*_2_, and *S*_3_ operated at 200 °C for the different target gases (propanol, acetone, and ethanol) under ambient air is reported. The resistance of the sensor decreases with the increasing concentration of gases (0–5000 ppm). It is observed that the 2 wt% CdS-doped SnO_2_ sensor showed the greatest response (78%), and is highly selective (44.6%) to propanol over acetone and ethanol with a fast response and recovery time. Thus, we conclude that the CdS doped-SnO_2_ thick film sensor is highly sensitive. Furthermore, it is more selective to propanol gas than the other target gases.

## Conflicts of interest

The authors declared that they have no competing interests or personal relationships that could have appeared to influence the work reported in this paper.

## Supplementary Material
